# Putative genome contamination has minimal impact on the GTDB taxonomy

**DOI:** 10.1099/mgen.0.001256

**Published:** 2024-05-29

**Authors:** Aaron J. Mussig, Pierre-Alain Chaumeil, Maria Chuvochina, Christian Rinke, Donovan H. Parks, Philip Hugenholtz

**Affiliations:** 1 The University of Queensland, School of Chemistry and Molecular Biosciences, Australian Centre for Ecogenomics, St Lucia, QLD, Australia

**Keywords:** genome contamination, GTDB, phylogeny, taxonomy

## Abstract

The Genome Taxonomy Database (GTDB) provides a species to domain classification of publicly available genomes based on average nucleotide identity (ANI) (for species) and a concatenated gene phylogeny normalized by evolutionary rates (for genus to phylum), which has been widely adopted by the scientific community. Here, we use the Genome UNClutterer (GUNC) software to identify putatively contaminated genomes in GTDB release 07-RS207. We found that GUNC reported 35,723 genomes as putatively contaminated, comprising 11.25 % of the 317,542 genomes in GTDB release 07-RS207. To assess the impact of this high level of inferred contamination on the delineation of taxa, we created ‘clean’ versions of the 34,846 putatively contaminated bacterial genomes by removing the most contaminated half. For each clean half, we re-calculated the ANI and concatenated gene phylogeny and found that only 77 (0.22 %) of the genomes were not consistent with their original classification. We conclude that the delineation of taxa in GTDB is robust to the putative contamination detected by GUNC.

**Impact Statement**

GTDB has become a widely adopted reference taxonomy for bacteria and archaea, often used to quantify diversity in metagenomic analyses of microbial communities. The artificial inflation of this reference taxonomy via contaminated genomes would substantially compromise the utility of GTDB. This study finds that putative contamination negligibly impacts the GTDB taxonomy, meaning that the resource can be reliably used as a standard for microbial diversity estimates.

## Data Summary

Scripts used in this study are available on GitHub https://github.com/aaronmussig/impact-of-contamination-on-taxonomy and the generated data are available on UQ eSpace: https://doi.org/10.48610/85c83e3

## Introduction

High-throughput sequencing and high-performance computing have been a boon for the exploration of microbial diversity by expediting the recovery of microbial genomes from isolates, environmental samples (metagenome-assembled genomes; MAGs) and single cells (single amplified genomes; SAGs) [1]. However, contamination may be introduced into genome sequences through a variety of sources including experimental and computational, which can compromise downstream analyses [2]. In particular, MAGs have been singled out as being at high risk of contamination as contigs putatively belonging to a single population are binned from a heterogeneous pool of metagenomic contigs [3]. However, even isolate genomes can suffer from contamination if they are not axenic or if reagent contaminants are sequenced and included in draft assemblies [4].

Consequently, many tools have been developed to try to detect and sometimes remove putative contamination from draft genome sequences. These include at least 18 tools of which six were recently benchmarked [2]. The Genome UNClutterer (GUNC) [5] was found to be one of the best performing tools with a low false-positive rate [2]. However, this study also highlighted the lack of reproducibility between different tools, and the difficulty in distinguishing natural biological processes such as horizontal gene transfer from contamination [2].

In the original GUNC study, it was suggested that genome contamination has artificially inflated diversity estimates in the Genome Taxonomy Database as ~3 % of species in GTDB release 05-RS95 were inferred to be entirely comprised of contaminated genomes [5]. Here, we critically examine the impact of putative genome contamination on taxon delineation in GTDB by systematically removing the most contaminated portions of affected genomes and reinferring the reference taxonomy. We find limited evidence for taxonomic inflation due to contamination and indeed lower than baseline changes from random removal of genomic segments.

## Methods

### Identifying putatively contaminated genomes

Nucleotide files for the 317, 542 genomes that passed quality control in GTDB release 07-RS207 [6] were obtained from the NCBI Assembly database release 207 [7]. Genes were called for each genome using Prodigal v2.6.3 [8] according to translation tables 4, 11 or 25 as specified in the GTDB metadata files (https://data.gtdb.ecogenomic.org/releases/release207/207.0/). Called genes for each genome were processed with GUNC v1.0.5 [5] using the GTDB 05-RS95 and ProGenomes 2.1 [9] DIAMOND reference databases provided with the GUNC software. GUNC results for these two databases were merged by inclusion of all failed genomes. If a genome was identified as contaminated with both reference databases, the result with the highest clade separation score (CSS) was used as the worst-case scenario for downstream analyses.

### Calculation of contig contamination scores

**Fig. 1.**
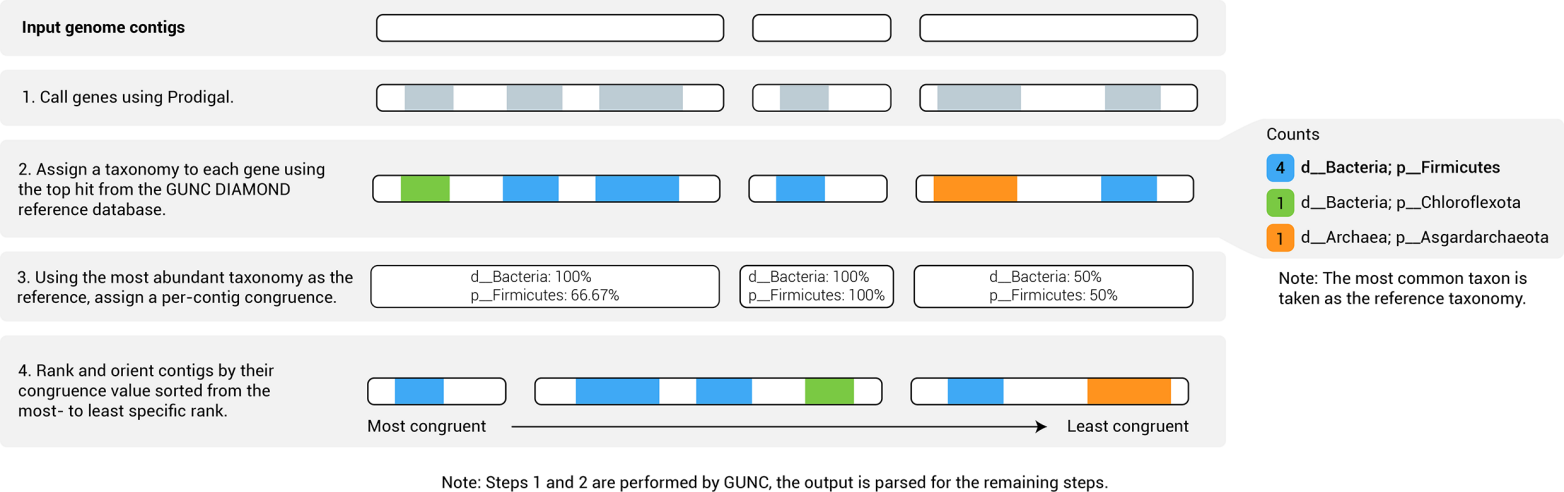
Calculation of GUNC-based contamination scores for individual genome contigs.

Since GUNC does not provide a contamination score for individual contigs in a draft genome, we developed a contig-based scoring system for GUNC-failed genomes (Fig. 1). First, a taxonomic assignment was determined for each genome by taking the most commonly inferred taxon at each rank across the GUNC-provided assignments across all genes. This genome-specific taxonomic assignment was then used as a reference taxonomy for establishing the classification congruence of each contig. The taxonomic assignment of each contig was determined by taking the majority vote at each rank of the GUNC-provided closest DIAMOND match to each gene on the contig. In the rare case of ties, the tied rank was not considered. The taxonomic assignment was then truncated to the genome-specific rank at which the largest CSS occurs, as identified by GUNC. For each contig, the proportion of genes that had a congruent assignment with the truncated rank specified above was determined. The contigs were then ordered by how much the contig-specific taxonomic assignment deviates from the genome-specific taxonomic assignment, from domain to the rank at which the largest CSS occurs. The greater the deviation the higher the contamination score (Fig. 1).

### Dividing GUNC-failed genomes by contig contamination scores

Contigs in each failed genome were ordered by their contamination score and the cleanest half of the genome (*i.e.* contigs with the lowest contamination scores) was retained for subsequent analysis. Removal of the contaminated half of each genome was based either on nucleotides or marker genes depending on the analysis (*see below*). In the case of nucleotides, exactly half of the total nt length of the genome was removed. If this required the division of a contig, the contig was oriented from most to least contaminated (Fig. 1 step 4) and the most contaminated end was pruned to the required length. In the case of marker genes, if half the marker gene count occurred within a contig, the contig was also ordered from most to least contaminated and the necessary number of marker genes removed from the contaminated end.

### Average nucleotide identity (ANI)-based analysis of clean genome halves

The clean halves of all failed genomes were assigned to their closest GTDB species representative in release GTDB 07-RS207 using a combination of Mash [10] and FastANI [11]. A reference database was first created using Mash v2.3 comprising the 62,291 bacterial species representatives in GTDB 07-RS207. All species representatives with >80 % ANI to each clean half were identified using the Mash parameters *k*=16, *s*=5000, *d*=0.2, and *v*=1.0. A second set of species representatives were obtained for each failed genome using their original classification, anticipating the possible movement of clean halves from their original position in the reference tree. This included all species representatives from the expected genus, and if this comprised fewer than 100 genomes, then all species representatives were included from the ranks of family to phylum until at least 100 additional genomes were added using this method. This provided sufficient phylogenetic context to detect misclassifications, balanced against computational requirements. FastANI v1.3 was run bidirectionally (-q and -r) against the union of the two sets of species representatives obtained for each failed genome to identify the closest match, taking the maximum ANI and alignment fraction (AF) as one result. Self-hits were excluded for failed genomes that were species representatives in GTDB 07-RS207 and the next best match was then considered.

Based on the closest FastANI hit, each failed genome was assigned to one of three categories using the species assignment criteria of ≥95 % ANI and ≥0.5 AF [6]: (i) *same species cluster* when the closest hit by ANI was the expected species representative and the species assignment criteria were satisfied, (ii) *changed species cluster* when a new representative genome was closest and the species assignment criteria were satisfied, and (iii) *new species cluster* when the closest hit by ANI did not satisfy the species assignment criteria.

To estimate background noise of the ANI analysis, we selected equivalently sized nucleotide subsets of genomes in GTDB 07-RS207 that passed the GUNC analysis. A random ordering of contigs was generated for each passed genome and the first half of the genome was retained for analysis. Datasets were then analysed as per the clean half dataset. This was repeated 10 times to determine the baseline distribution of taxonomic changes on passed genomes.

### Tree-based analysis of clean genome halves

Aligned marker genes from the clean halves of the 4,525 failed genomes that are used as representatives of species clusters in GTDB 07-RS207 were obtained from the GTDB website (https://data.gtdb.ecogenomic.org/releases/release207/207.0/genomic_files_reps/bac120_msa_marker_genes_reps_r207.tar.gz). For each genome the clean set of marker genes was concatenated and aligned to the bac120 multiple sequence alignment (MSA) [12]. These 4,525 alignments were then substituted for their original alignments in the GTDB 07-RS207 bacterial species representative MSA comprising 62, 291 sequences. The modified MSA was then masked using the standard GTDB filter (https://data.gtdb.ecogenomic.org/releases/release207/207.0/auxillary_files/bac120_msa_mask_r207.txt), and a maximum-likelihood tree inferred with FastTree v2.1.10 [13] using the WAG [14] model. The tree was bootstrapped 100 times using GenomeTreeTk v0.1.8 [15] and decorated with the GTDB 07-RS207 bacterial taxonomy using PhyloRank v0.1.12 [16].

Congruency of GTDB classifications between clean and original failed genomes was scored by comparing the taxonomy string of each genome derived from the inferred tree against its original GTDB 07-RS207 classification. This was performed from phylum to genus, with the highest ranked incongruent name being recorded. All incongruencies were manually checked by comparison of tree topologies in ARB [17].

### Identification of grossly contaminated genomes

Genomes with contamination from distantly related organisms belonging to different families or higher ranks were identified by processing contaminated halves of all failed genomes using ANI- and tree-based analysis as described above. The taxonomic strings for the clean and contaminated halves of each genome were compared to each other as described above to estimate the degree of contamination.

## Results and discussion

### Identification of putatively contaminated genomes

**Fig. 2.**
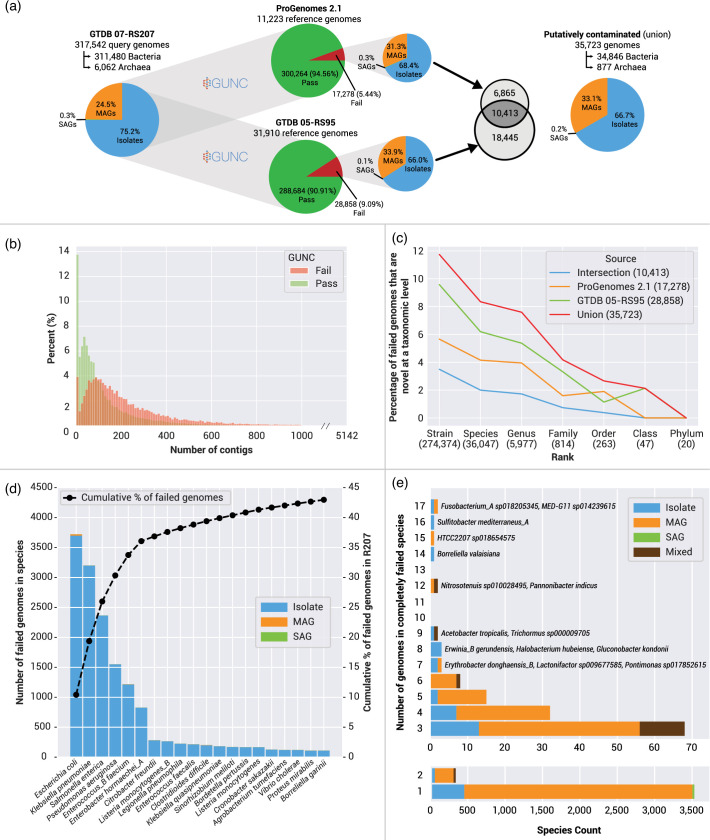
Application of GUNC to bacterial and archaeal genomes in GTDB release 07-RS207. (**a**) Summary of passed and failed genomes using the two reference databases available through GUNC. (**b**) Histogram of percentage of passed and failed genomes as a function of contig number. (**c**) Failed genomes as a function of taxonomic novelty, showing that failure rates decline with increasing taxonomic novelty. (**d**) Top 20 GTDB-defined species with the highest number of failed genomes (left *Y*-axis) representing almost half of all failed genomes (right *Y-*axis). (**e**) GTDB-defined species comprised exclusively of failed genomes as a function of the number of representatives in the species and genome type.

To assess the impact of genome contamination on the GTDB taxonomy, we first analysed the complete GTDB 07-RS207 dataset with GUNC 1.0.5 using the provided ProGenomes (2.1) and GTDB (05-RS95) reference databases. Of the 317,542 07-RS207 query genomes, 35,723 (11.25 %) were flagged as putatively contaminated (failed) using the ProGenomes and GTDB reference databases (Fig. 2a). The proportion of failed genome types (isolates, MAGs, SAGs) broadly reflected the proportion of input genomes, with MAGs being over-represented; 25 % of input genomes, 33 % of failed genomes (Fig. 2a). Failed genomes were notably more fragmented than passed genomes, with a skew towards increased contig count (Fig. 2b). This is consistent with the expectation that more fragmented genomes have a higher chance of contamination, particularly in the case of MAGs [3].

We then divided the input genomes by their degree of taxonomic novelty according to GTDB 07-RS207 (noting that we use the taxonomy of that release throughout this study). For example, there are 20 genomes that were the sole representatives of phyla in 07-RS207, of which none failed the GUNC analysis, and 274,374 genomes that are only novel at the strain level (*i.e.* belong to a known species), of which 32,218 (11.7 %) failed (Fig. 2c). These strain-level failures comprise 90 % of all failed genomes and are biased towards the most represented species in GTDB including *Escherichia coli* (3,721 of 26, 859 genomes classified as *E. coli*), *Klebsiella pneumoniae* (3,201 of 11,294), and *Salmonella enterica* (2,365 of 12,285), which are all dominated by isolate genomes (Fig. 2d). If this is a true representation of species-level contamination, it has important implications for pangenome analyses of microbial species in that the size of the species accessory genome will be artificially inflated and may give the appearance of an open pangenome [18,19]. However, the GUNC authors themselves caution against over-interpretation of contamination estimates at the species and strain level as the method becomes less precise between closely related lineages [5]. Conversely, detection of contamination in phylogenetically novel genomes, such as the 20 bacterial phyla sole representatives, may be underestimated by GUNC as the gene-wise taxonomic classifications may lack close hits in the reference databases. Regardless of these potential limitations, the chances of a genome being failed decreased as a function of taxonomic novelty (*i.e.* from strain to phylum) and increased as a function of the GUNC reference database size (*i.e.* the larger GTDB 05-RS95 database identified more putative chimeric genomes). Furthermore, the intersection of the failed genomes between the reference databases was only 29.2 % (10,413 of the combined 35,723) and this intersection decreased with increasing taxonomic novelty of the genomes (Fig. 2c). This highlights the dependency of GUNC on the reference database used and the taxonomic novelty of the query genomes [5]. Disturbingly, 6 % (3,958) of species defined in 07-RS207 were comprised exclusively of failed genomes (Fig. 2e). This represents a substantial erroneous inflation of the number of taxa in GTDB release 07-RS207 (Table 1).

**Table 1.** Number of taxa by rank in GTDB release 07-RS207 that were comprised entirely of GUNC-failed genomes. The number in brackets represents the percent inflation (no. failed / total – failed) at each rank that would be caused by inclusion of these putatively spurious taxa as determined by GUNC

	Phylum	Class	Order	Family	Genus	Species
Archaea	0 (0.0)	0 (0.0)	1 (0.8)	4 (0.9)	34 (2.6)	257 (8.1)
Bacteria	0 (0.0)	1 (0.2)	7 (0.5)	43 (1.2)	532 (3.6)	3701 (6.3)
**Total**	**0** (0.0)	**1** (0.2)	**8** (0.5)	**47** (1.2)	**566** (3.5)	**3958** (6.4)

### Division of GUNC-failed genomes into ‘clean’ and ‘contaminant’ halves

**Fig. 3.**
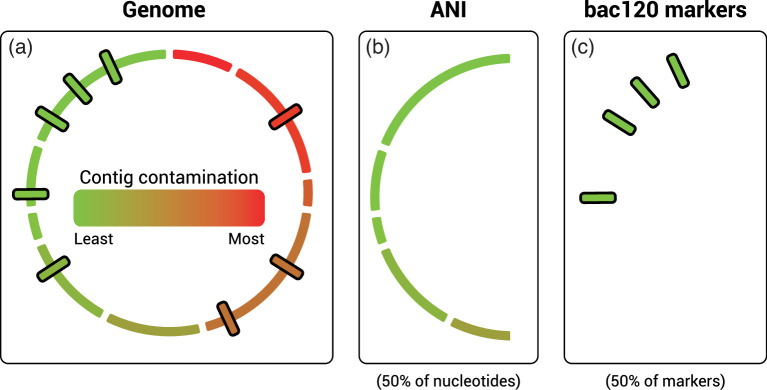
Graphical representation of halving putatively contaminated genomes (**a**) by nucleotide or marker gene content. Contigs in failed draft genomes were first ordered by a GUNC-derived contamination score (Fig. 1) from most to least putatively contaminated. 50 % of nucleotides (**b**) or marker genes (**c**) were then removed from the most putatively contaminated contigs.

To assess the effect of putative contamination on the GTDB taxonomy, we focused our attention on the bacteria with the expectation that contamination affects bacterial and archaeal genomes in the same way. We began by dividing each failed bacterial genome into a ‘clean half’ and a ‘contaminant half’ on the presumption that contamination should comprise no more than 50 % of the total genome. Genomes with greater than this level of contamination are unlikely to pass standard GTDB quality controls, specifically CheckM contamination <10 %. GUNC currently scores contamination on a gene-by-gene basis [5]. These data were used to rank contigs within each of the 34,846 failed bacterial genomes by their degree of contamination (Fig. 1). For each genome, contigs were then ordered from most to least contaminated based on the per contig score and then divided into clean and contaminant halves (*directed removal,* Fig. 3). In line with common usage [20], the GTDB uses a 95 % average nucleotide identity (ANI) threshold to define species [12] and a concatenated single-copy marker gene (bac120) tree to define the ranks of genus to phylum. Therefore, we recapitulated this methodological approach to assess the effect of pruning putatively contaminated portions of failed genomes. For ANI analysis, the contaminant half of the genome was removed based on nucleotides (Fig. 3b), and for marker gene analysis, genomes were divided based on bac120 markers (Fig. 3c). Consistent with the expectation that this process removes contamination, CheckM contamination scores decreased from 2.236±2.044 % for the full marker set, to 0.004±0.056 % for the clean 50 % marker set. Since halving the genomes could result in loss of taxonomic signal, we included GUNC-passed genomes, randomly pruned to the same extent (*random removal*), as controls for the ANI-based analysis to establish a baseline of potential taxonomic changes due to factors other than genome contamination, *e.g.* loss of phylogenetic signal [21] or change in ANI alignment fraction [22]. A baseline analysis was not performed for the *de novo* tree inference as very few misclassifications were found by directed removal of bac120 marker genes (*see below*).

### Evaluation of putative genome contamination on ANI-based species classifications

**Fig. 4.**
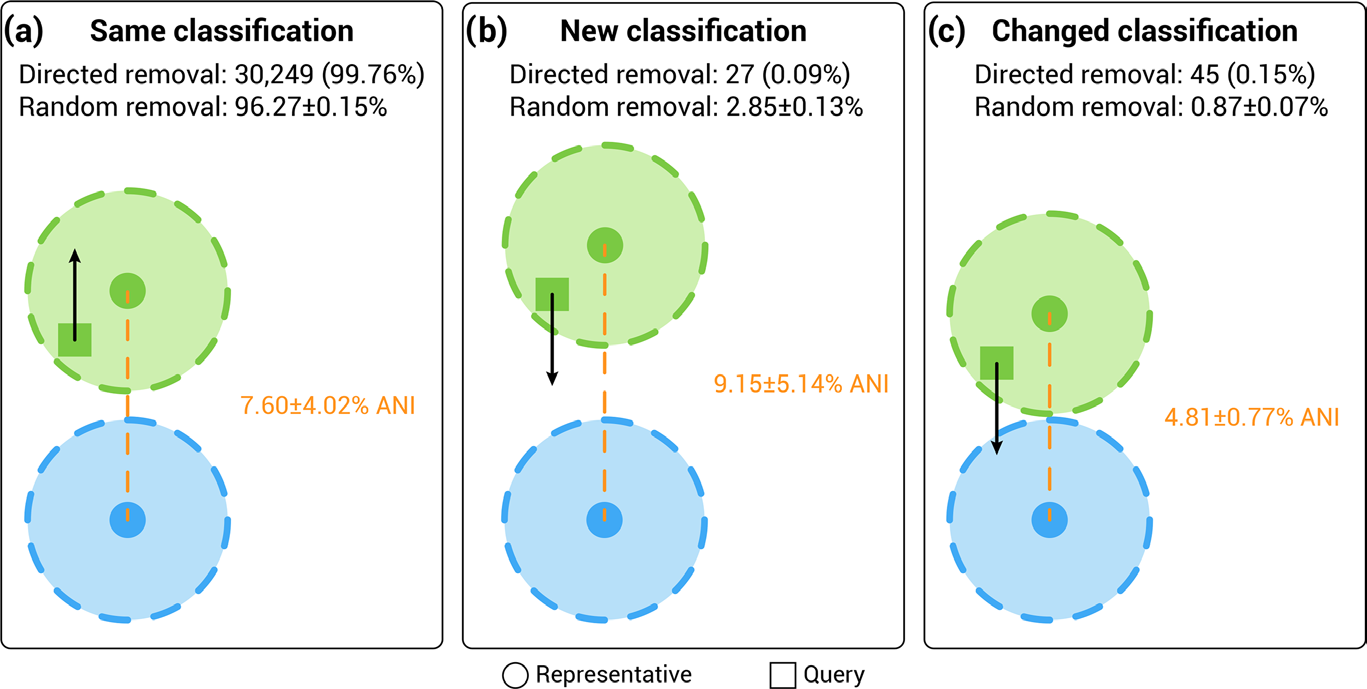
Summary of ANI-based taxonomic congruency analysis between the original and clean half classifications of the failed 07-RS207 genomes showing that the great majority of genomes retained the same classification after directed removal. For comparison, the same metrics are shown for randomly selected passed genomes (random removal) to estimate background noise in the analysis. (**a**) No change in classification of failed genomes as graphically depicted by the movement (black arrow) of the failed genome (green square) staying within the ANI radius (≥95 %) of the species representative (green circle). The average ANI distance between the original species (green) and closest neighbouring species (blue) is shown as a dashed orange line. (**b**) New classifications after removal of contaminated half due to movement of the failed genome outside the ANI radius of the original species. (**c**) Changed classifications due to movement of failed genomes into the ANI radius of a neighbouring species, noting that this occurs in more densely populated genera with lower inter-species representative distances.

For the ANI analysis, clean halves were compared to the 62,291 bacterial species representatives in 07-RS207 with the exception of 4,525 failed bacterial genomes that were species representatives in that release. These 4,525 genomes were excluded because they would have 100 % identity to their complete species representative versions, and were instead assessed for their potential impact on the GTDB taxonomy via the bac120 phylogeny analysis (*see below*). We anticipated three possible outcomes of the ANI analysis; the clean half (i) remained within the radius of its original species representative (*same classification*), (ii) moved out of the radius (*new classification*) or (iii) moved into the radius of another species representative (*changed classification*; Fig. 4). Of the 30,321 failed bacterial genomes that were not species representatives, 30,249 (99.76 %) clean halves had the same classification as the complete genome with only 45 (0.15 %) being assigned to a different species of the same genus (Fig. 4c; Table S1, available in the online version of this article). This indicates that half a genome is sufficient to ensure accurate ANI calculations as previously demonstrated [23]. A further 27 (0.09 %) had a new classification (Fig. 4b; Table S1), and in these few instances the distance to the nearest neighbouring species was greater than the equivalent distance in the same or changed species (Fig. 4). We attribute the 45 changed classifications to species hopping within densely populated genera. Such regions of the tree have tight interspecies boundaries with the average distance between representatives of the closest neighbouring species being 4.81±0.77 % for genomes that changed classification compared to 7.60±4.02 % for genomes that retained the same classifications after pruning, or 9.15±5.14 % for new classifications. Overall changes in classification for the random removal control groups were higher than in the test group, 0.87±0.07 % vs 0.15 % for changed classifications and 2.85±0.13 % vs 0.09 % for new classifications (Fig. 4). We attribute this difference to an increased likelihood of randomly removing genomic contigs that contributed to the original ANI calculation used to classify the genome. By contrast, putatively contaminated contigs removed by directed removal would be less likely to contribute to the ANI calculation since ANI is only calculated over homologous regions (*see examples below*). This demonstrates that for the great majority of genomes, putative contamination made no difference to the ANI-based species classification used by GTDB.

### Evaluation of putative genome contamination on genus to domain classifications using *de novo* bacterial tree inference

For the *de novo* tree-based analysis, we used the GTDB bac120 marker set to infer a reference tree of the 62,291 bacterial species representatives in 07-RS207, but only included the clean halves of the 4,525 failed species representatives based on marker counts (Fig. 3c). Changes in classification between the original (complete) and pruned genomes at genus or higher ranks (depending on the level of taxonomic novelty of the species representative; Fig. 1c) were determined using PhyloRank decorate and manual comparison of tree topologies (*see Methods*). Only five of the 4,525 (0.11 %) failed species representatives were taxonomically incongruent via tree-based analysis, *i.e.* the immediate higher rank was different to the original classification; three were classified as a different genus in the same family, one as a different family in the same order, and one as a different class in the same phylum (Table S1).

### Classification changes depend on taxonomic relatedness of the contamination

**Fig. 5.**
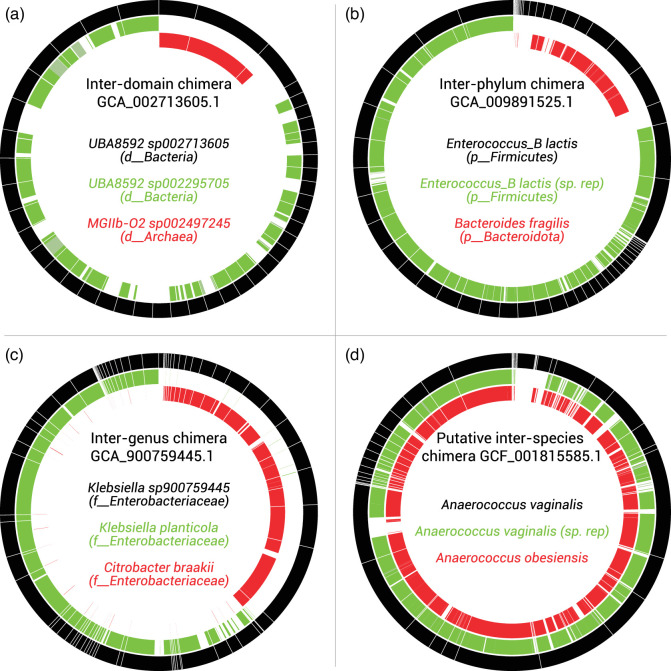
Four putatively chimeric genomes (failed by GUNC) are shown in black with contigs ordered from most to least contaminated clockwise from the top of each ring as per Fig. 3. Closest matched genomes (not failed by GUNC) are shown as red and green inner rings in regions with ≥85 % ANI to the chimeric genome. Labels are coloured accordingly and the parent taxon of each genome at or above the level of predicted contamination is shown in brackets for panels **a** to **c** (d__=domain, p__=phylum, f__=family). Grossly contaminated genomes (inter-family contamination or higher, panels (**a**) and (**b**)) retained the same classification (Fig. 4a), whereas the inter-genus chimaera (**c**) and putative inter-species chimaera (**d**) resulted in a new and changed classification, respectively.

As detailed above, a vanishingly small number of putatively contaminated genomes resulted in a change to their GTDB classification according to both ANI and bac120-based analyses. These 47 MAGs and 30 isolate genomes represent at maximum 71 spurious species in release 07-RS207 (Table S1). This constitutes only a 0.1 % inflation of the number of species in 07-RS207 with no inflation at higher ranks, much lower than the original inflation estimates based on the GUNC results (Table 1). Furthermore, some of these putatively spurious species may in fact be due to methodological artefacts or biological processes such as recombination, rather than genome contamination *per se* as evidenced by a background of similar low level (inter-species) misclassifications in the control set of randomly pruned genomes that passed the GUNC analysis (Fig. 4). By contrast, none of the clean halves of 43 grossly contaminated genomes (inter-family contamination or above) detected via analysis of the contaminant halves of failed genomes (*see Methods*), were misclassified (Table S2). Note that 34 of these genomes were species representatives that could only be classified to their level of taxonomic novelty in the bac120 tree, but would not result in taxonomic inflation. Eleven of these 43 genomes have since been flagged as contaminated by NCBI (Table S2). Four examples of putatively contaminated genomes that resulted in, or did not result in, classification changes are shown in Fig. 5 to illustrate how contamination can affect GTDB classifications. Extreme contamination of genomes such as the inter-domain MAG chimaera, *UBA8592 sp002713605* (GCA_002713605.1; Fig. 5a), or inter-phylum isolate chimaera *Enterococcus_B lactis* (GCA_009891525.1; Fig. 5b) resulted in no change to the GTDB classification because the contaminating portions did not directly contribute to either the ANI or bac120 analysis, *e.g.* the archaeal contaminant in *UBA8592 sp002713605* lacks bacterial marker genes (Fig. 5). Conversely, contamination between more closely related genomes can result in a classification change because the contaminating sequence is close enough to the primary genome to influence both the ANI and bac120 analysis. For example, a spurious *Klebsiella* species (*Klebsiella* sp900759445; GCA_900759445.1) was formed due to contamination of *Klebsiella planticola* with *Citrobacter braakii* (inter-genus contamination), and *Anaerococcus vaginalis* (GCF_001815585.1) changed classification to the sister species *A. obesiensis* following removal of putative *A. vaginalis* contamination (Fig. 5). Note however, that this latter case is marginal and may reflect methodological artefacts, such as species hopping in densely populated genera or even biological processes between the species such as homologous recombination [24].

In summary, we have shown that putatively contaminated genomes detected by GUNC have minimal impact on the GTDB taxonomy with an estimated 0.1 % inflation of the number of species in release 07-RS207, which is below background for the ANI-based method used in this study (Fig. 4). This is not to say that GUNC-failed genomes are not contaminated, but rather that the contamination is insufficient to alter the GTDB classification in the great majority of cases, which was our primary concern as a taxonomic resource. Our findings also support the use of genome sequences as type material for the proposed SeqCode nomenclature [25,26] because taxonomic assignments are robust to potential contamination and incompleteness. As genome quality continues to improve with long-read sequencing technologies producing more complete genomes [27,28], genome contamination will become less of an issue for comparative genomics. However, even at the present level of genome quality, we demonstrate that contamination is not an issue for the genome-based classification implemented in GTDB.

## supplementary material

10.1099/mgen.0.001256 Table S1.

## Abbreviations

AF: alignment fraction
ANI: average nucleotide identity
CSS: clade separation score
GTDB: Genome Taxonomy Database
GUNC: Genome UNClutterer
MAG: metagenome-assembled genome
MSA: multiple sequence alignment
SAG: single amplified genome
